# Working Memory in the Processing of the Iowa Gambling Task: An Individual Differences Approach

**DOI:** 10.1371/journal.pone.0081498

**Published:** 2013-11-20

**Authors:** Virginie Bagneux, Noémylle Thomassin, Corentin Gonthier, Jean-Luc Roulin

**Affiliations:** 1 Laboratoire Inter-universitaire de Psychologie, University of Savoy, Chambéry, France; 2 Laboratoire de Psychologie et NeuroCognition, University of Savoy, Chambéry, France; Centre national de la recherche scientifique, France

## Abstract

The Iowa Gambling Task (IGT) is a sequential learning task in which participants develop a tendency towards advantageous options arising from the outcomes associated with their previous decisions. The role of working memory in this complex task has been largely debated in the literature. On one hand, low working memory resources lead to a decrease in the number of advantageous decisions and make a significant part of participants unable to report explicitly which options are the most profitable. On the other hand, several studies have shown no contribution of working memory to the IGT decision patterns. In order to investigate this apparent incompatibility of results, we used an individual differences approach, which has proven an effective method to investigate the role of working memory in cognition. We compared the IGT decision patterns of participants as a function of their working memory capacity (WMC). As expected, contrary to low WMC participants, high WMC participants developed a tendency towards advantageous decisions. These findings lead us to discuss the role of WMC in decision making tasks.

## Introduction

In the Iowa Gambling Task (IGT) [1], participants have to make a set of decisions under uncertainty, because probabilities associated with each decision outcome are unknown and unpredictable. Players have to pick cards from four different decks during a set of 100 trials. Two disadvantageous decks offer the opportunity of making large gains but greater losses, whereas two advantageous decks offer smaller gains but also smaller losses. As a result, the advantageous decks are more profitable in the long term.

The IGT was originally developed to assess decision making impairments in patients with damage to the ventromedial prefrontal cortex. These patients do not develop a tendency towards advantageous options in the task, contrary to non-clinical participants [1,2]. Bechara and Damasio observed four distinct periods of decision making in the IGT [3]: 1) normal participants pick cards before they encounter any negative outcomes (pre-punishment period); 2) they go on and receive punishments without having any idea of what is happening (pre-hunch period); 3) around 50 pickings, participants have enough knowledge of the task to begin expressing hunches about which decks are more advantageous, but without being certain (hunch period) [4]; 4) they clearly know which decks are advantageous and disadvantageous (conceptual period). 

Interestingly, decision making in the IGT seems to rely on "hunches" or emotional cues for the choice of advantageous decks. Seeing as there is a wide range of rewards and punishments and the associated probabilities of these outcomes are unpredictable by participants, and seeing as cognitive resources are limited, it does not seem possible to explicitly calculate the net gains/losses for each option. This led Bechara and Damasio to conclude that normal participants may take into account the emotional cues shaped by the association between reward, punishment and the elicited emotion (i.e., somatic markers) to develop a tendency towards advantageous decisions [3]. These emotional cues would subsequently guide decision-making. In other words, participants would strongly rely on their hunches or emotional cues to make advantageous decisions, as opposed to using an explicit knowledge of the relative values of the decks [5].

### Working Memory and the Iowa Gambling Task

This somatic markers framework in its initial form gives a central role to emotional cues in the IGT, with little role for explicit cognitive processes [2,6]. If explicit knowledge of the outcomes of each deck does not factor in the task, then working memory (WM), in particular, should not play an obvious role in the IGT [1]. However, the same authors have also argued that WM and IGT performance may hold asymmetric relationships: impaired performance on the IGT may occur without any WM impairment, but WM impairment could lead to difficulties in decision making [3,7]. In this view, the WM system could be required to store options and scenarios about the task; somatic markers would act by biasing the representations stored in working memory [3]. These two accounts of the somatic markers framework seem difficult to reconcile; however, the possibility of a WM involvement in the IGT is far from trivial, as it directly questions the emotion-based nature of the task.

What do empirical results tell us about the relationship between WM and the IGT? Several studies have assessed the role of WM with various methodologies; these works give contrasted pictures. Firstly, a few studies have addressed the question of WM involvement in the IGT at the neurological level. In some patients with a prefrontal lesion, WM and decision making deficits seem to be separable [6]: patients with damage to the ventromedial prefrontal cortex show a normal WM but an impaired IGT decision pattern, whereas patients with damage to the dorsolateral prefrontal cortex show an impaired WM but an advantageous IGT decision pattern. These results prompted the authors to conclude that WM and decision-making are doubly-dissociated. However, more recent pathological studies in patients with damage to the prefrontal cortex [8] and in patients with substance dependence [9] question this double dissociation: for example, patients with a dorsolateral prefrontal lesion have been observed to fail the IGT [8,10]. Moreover, a recent fMRI study observed activations both in the dorsolateral and in the ventromedial prefrontal cortex during the course of the IGT [11], suggesting that the task involves at least some WM demands.

A second line of research tried to apply a load to WM, with the idea that if the IGT requires WM, then taxing WM should lead to less advantageous decision making on the IGT. Two such studies failed to observe an effect of WM load on decision making under normal IGT conditions [12,13], whereas two other studies observed an effect of WM load [14,15]. Moreover, it has been observed that participants realizing an interfering secondary task - supposed to tax WM - during the first 100 trials of the IGT show a lower number of advantageous decisions in the IGT [16] – while the same interfering task has no effect on decision making after the 100^th^ trial. This result suggests WM may be necessary to efficiently process the outcomes of decisions at the beginning of the task in order to develop hunches.

Thirdly, other authors have addressed the question of WM involvement by means of modified versions of the IGT. A first study used a version of the IGT relying only on WM with no contribution of emotional cues, the Fire-fighters Task [17]. In this task, the participants had to evaluate fire-fighters on the basis of their actions, which could be positive, negative or neutral. The task used the same probabilistic reinforcements than the IGT. The authors argued that the absence of any direct reward or punishment for participants on each trial would prevent the use of emotional cues and would constrain participants to rely specifically on WM. The Fire-fighters Task proved to be too complex to be resolved using solely the relative values of the fire-fighters actions, which suggests that in the absence of emotional cues, WM is not sufficient to complete a version of the IGT. Another study tried to randomize the spatial positions of the four decks in the IGT across trials, thereby increasing the WM requirements in the task [18]. In this situation, only participants performing under a low WM load – compared to a high WM load – made advantageous choices. It suggests that WM was required to successfully perform this version of the IGT. WM load was also disruptive of decision patterns in a reversed version of the IGT, where the advantageous decks yield a high immediate punishment but a higher delayed reward [19]. More precisely, participants constrained by a high WM load made a significantly greater number of disadvantageous decisions.

Overall, these three lines of studies give a mixed picture about WM involvement in decision making on the IGT. Some studies observe no contribution of WM to the IGT, while others suggest such a contribution might exist under certain conditions.

### The Individual Differences Approach

Interestingly, there is another method of studying the role of WM: the individual differences approach. An abundant literature evidences that there exist individual differences in WM capacity (WMC) in non-pathological, adult participants. One of the more robust experimental results in this field is that individual differences in WMC are related to a great variety of complex cognitive abilities [20], which could very well include decision making. In other words, it is also possible to examine WM involvement in the IGT by using the intrinsic individual differences that exist independently of any experimental manipulation.

A single meta-analysis was conducted with an individual differences approach to investigate the link between the IGT and several executive functions, including WM [21]. The authors found no significant correlation between WM and performance on the IGT; overall, a median value of *r* = .06 was calculated. The authors concluded that performance on the IGT and measures of WM seem to be relatively dissociated. However, among the fifteen studies included in the analysis, only two included non-clinical adult samples, which raises the question of the representativeness of this meta-analysis; these two non-clinical studies gave opposite results [22,23]. Moreover, one of these two studies used the forward digit span as a WMC measure; in fact, the forward digit span is a simple span task that measures short term memory rather than WM [24]; [25]. This distinction is important because short-term memory and WM are two separable constructs that bear different relationships to high-level cognition. In summary, the literature shows a critical lack of reliable data on the link between WM and the IGT obtained with the individual differences approach.

### Overview

The goal of the current study was to use the methods of individual differences research to investigate the role of WMC in the IGT, using a classical WM task with a non-clinical adult sample. This approach offers two main advantages: firstly, it allows for the validation of prior findings in the IGT literature with a different method; secondly, if WM does truly contribute to IGT decision pattern, then this contribution might prove more sensitive to individual differences in WMC than it does to experimental manipulations of the IGT or WM load. Indeed, the observed discrepancies in the literature may be in part attributable to limitations of the experimental methods. For example, the lack of a WM loading effect observed in the study of Turnbull and colleagues could be explained by the lack of external time constraints over the participants’ random production [12,16]; [18], so that participants could slow their RNG production and limit their WM load when the WM demands in the IGT were increased.

In line with the individual differences approach, we elected to examine the IGT decision patterns as a function of WMC. If WM is involved in the IGT decision patterns, then high WMC participants should develop a stronger tendency towards advantageous decisions than low WMC participants.

It is possible that WM is not sufficient to process the IGT independently of emotional cues, as suggested by the Fire-fighters Task [17], but that it is necessary to process the outcomes of previous decisions to develop hunches, as suggested by Stocco and Fum [16]. The "hunch period" of the IGT appears between the 40^th^ and 60^th^ trial [3]; Wagar and Dixon noted that the optimal strategy becomes advantageous in the IGT between the 34^th^ and the 56^th^ trial (i.e., when the preference for advantageous options arises because a large number of punishments has been encountered in the bad decks) [4]. For most authors, normal participants have enough information about the task to begin expressing hunches (emotional cues) about which options are the most advantageous after about 50 trials [2,4]. If it is truly the case that WM plays a role in the emergence of hunches, then a correlation between WM and the IGT performance should appear after a number of trials sufficient for participants to develop hunches. In that case, a higher WMC should be related to more advantageous decisions only from the third block of decisions onward.

## Methods

### 1: Ethics statement

The study protocol was approved by the ethical committee of the University of Savoy, in Chambéry. All participants gave their informed consent prior to the experimental session. The purpose of the experiment was clearly stated in the information notice.

### 2: Participants

Ninety-nine students from the University of Savoy (France) volunteered to participate in exchange for course credit. Ten participants were excluded from data analysis because they failed the working memory task (see below), three participants were excluded following an outliers analysis and three participants were excluded because of a history of psychiatric or neurologic disorders. The final sample included 83 participants (13 males). The mean age was 19.37 years (SD = 1.53). All participants were first-year psychology students, and none of them had previously completed the IGT.

### 3: Material

#### Modified Iowa Gambling Task

For the current study, we used a modified version of the IGT (mIGT. This version of the task was used because the standard version of the IGT presents validity problems in a French sample; see Bagneux, Font and Bollon for details [26]). The IGT was converted into a computerized simulation of stock-market investment; the task was exactly the same as in the original IGT, except that the interface was different. The study was presented as being carried out in collaboration with a fictional private company, and consisting of giving investment advice in a business context, within a computerized simulation of the stock-market. Each participant was given a fictitious 2000€ to invest in one of four companies. These four companies corresponded to the four decks in the original IGT; they were described as equivalent and identified only with a non-significant trigram and logo. 

The participants were asked to make a series of investment choices with the aim of earning as much money as possible. Each choice generated a profit or a loss. After each trial, a new screen displayed the outcome of the participant’s choice, along with the running total of money, for 3000 milliseconds. As in the original task, the amount and frequency of losses varied [1]. Choosing mostly disadvantageous companies would lead to an overall loss (250€ for every ten choices), whereas choosing advantageous companies would lead to an overall gain (250€ for every ten choices). The simulation was stopped after 100 trials but participants did not know beforehand how many choices they would have to make. As a post-test question at the end of the task, the participants were asked which company they thought was the most profitable.

#### Working Memory Tasks

Working memory was assessed with three complex span tasks: the reading span, symmetry span and operation span [27]. These tasks are commonly used to assess WMC in individual differences research; taken together, they provide a better estimate of WMC than a single task would [27]. All three tasks relied on the same procedure: in each trial, the participant had to remember a short series of stimuli while completing a processing task between the presentations of each stimulus in the series.

 Each trial started with a fixation cross followed by a processing task. The participant had to answer the processing task to move on. This task was followed by a to-be-remembered stimulus presented for 800ms, followed by a 1000ms delay, then a new processing task, and so on. At the end of a series, the participant had to recall all to-be-remembered stimuli. The length of the series ranged from 3 to 8, with 2 trials per level of difficulty. The trials were presented in a pseudo-random order, so that participants couldn't anticipate the length of the next trial.

 In the reading span task, the to-be-remembered stimuli were digits (1-9), and the processing task required the participants to decide whether short sentences were correct. In the symmetry span task, participants had to remember the position of squares in a 4x4 matrix, and to decide whether geometrical shapes were symmetrical. In the operation span task, participants were to remember consonants and decide whether the results of math operations were correct.

The WMC score on each complex span task was calculated as the proportion of correctly recalled stimuli (partial-credit load scoring) [27]. The three individual recall scores were then standardized and averaged to obtain a global WMC measure. Participants with accuracy lower than 75% on one of the processing tasks were excluded from data analysis. 

### 4: Procedure

To keep participants from suspecting a link between the mIGT and the working memory task, the two tasks were presented to participants as two distinct experiments. Participants carried out the mIGT first, followed by the three working memory subtests. Participants were fully debriefed at the end of the experimental session; the whole session lasted approximately 45 minutes.

## Results

### 1: Analysis procedure

In accordance with conventional analyses of IGT scores [3], the 100 trials were divided into five blocks of twenty choices. The net score for each block was calculated by subtracting the number of advantageous decisions from the number of disadvantageous decisions [(C+D)-(A+B)]. A net score above zero implied an advantageous set of decisions, whereas a net score below zero implied a disadvantageous set of decisions. 

### 2: Decision making on the mIGT as a Function of Working Memory

**Figure 1:**
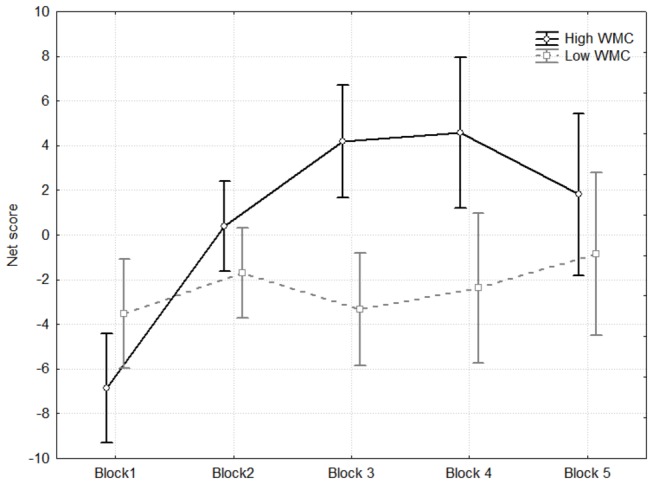
Net scores for each block of 20 trials as a function of working memory capacity. Net scores above zero indicate an advantageous set of decisions. WMC was treated as a categorical variable for easier interpretation; the low span group (*N* = 21) and high span group (*N* = 21) include participants falling in the lower and upper quartiles of the WMC distribution, respectively. Error bars represent 95% confidence intervals.

The analysis used the general linear model; it was a 5 (trial block) x WMC design. The results showed a main effect of WMC, *F*(1, 81) = 15.77, MSE = 70.72, *p* < .001; overall, global net scores increased with WMC. There was also a main effect of trial block, *F*(3.50, 283.48 [Greenhouse-Geisser correction]) = 15.36, MSE = 33.91, *p* < .001; performance increased over the course of the task. Finally, there was an interaction between WMC and trial block, *F*(3.50, 283.48 [Greenhouse-Geisser correction]) = 4.69, MSE = 33.91, *p* < .001 (Figure 1).

In order to comprehensively analyze the results, we tested whether decision patterns were significantly different as a function of WMC for each block separately. A high working memory was not related to more advantageous decisions in either Block 1, r(83) = -.03, *p* > .20, , or Block 2, r(83) = .07, *p* > .20. In other words, no differences emerged in decision making as a function of WMC before the hunch period. As expected, a higher WMC was related to more advantageous choices in Block 3, r(83) = .48, *p* < .001, and Block 4, r(83) = .37, *p* < .001. Unexpectedly, the net scores of the two WMC groups no longer differed in Block 5, r(83) = .17, *p* = .12.

### 3: Complementary analyses

The responses to the post-test question were analyzed with a logistic regression. Overall, a high WMC increased the probability to identify one of the two advantageous decks as being more profitable, χ^2^(78) = 5.08, *p* = .02. Of the 27 participants failing to correctly identify one of the advantageous companies, only three were in the upper quartile of the WMC distribution, suggesting that high span participants were more likely to develop explicit knowledge about the task. A post-hoc analysis indicated that the performance of participants falling in the upper quartile of the WMC distribution significantly increased from the first block to the last block of the task, *F*(1, 20) = 21.93, *p* = .001; this was not the case for participants in the lower quartile, *F*(1, 20) = 1.17, *p* = .30.

## Discussion and Conclusion

 The present study investigated whether decision making in the IGT relies on WM. To this end, we used an individual differences approach to test whether differences in WMC are related to differences in the pattern of results on the mIGT. As expected, a high WMC was associated with more advantageous decision patterns. This relationship appeared from the hunch period onward (third block of trials). These findings support previous results indicating a WM involvement in the IGT decision patterns [14,15,18,19]. In complement to the usual method of applying a load to working memory in order to test its relationship with the IGT, our study shows that individual differences in WMC are associated with different mIGT decision patterns regardless of any experimental manipulation.

A surprising result in classical studies of the IGT is that 30% of control participants do not reach the conceptual period, even though they manage to make advantageous decisions [3] (p. 348). Interestingly, the same proportion is observed in our results, and the probability of explicitly understanding which options are more advantageous is predicted by WMC. In other words, participants with a high WMC are more likely to reach the conceptual period. This suggests that individual differences in working memory could explain why some participants do not reach the conceptual period: these participants may be those with a low WMC.

One unexpected aspect of our results is the lack of a significant correlation between WMC and the mIGT net scores for the last block of decisions. This lack of a difference seems to indicate a change in participants’ decision-making over the course of the task. It is unlikely that this change is due to low WMC participants developing a tendency towards advantageous choices over the course of the task, as their performance did not significantly improve from the first to the last block. One possible explanation could be that high WMC participants tend to develop a propensity for risk seeking after they reach the conceptual period in the IGT. Several studies showed that risky selections in the IGT can reflect deliberative risk-taking after the development of explicit knowledge about the task rules, rather than a failure to recognize risk [28,29], it is possible that high WMC participants reached the conceptual period of the IGT after the first 80 trials and went on to engage in deliberative risk-taking.

As to the reason why high span participants make more advantageous decisions than low span participants, the question remains open. A first possibility is that low WMC participants take less advantageous decisions because they do not develop hunches about the values of the different decks. In some accounts of the somatic markers hypothesis, relying on hunches is the only way to make advantageous choices in the IGT [2,6]. Several clues point to an impairment of hunches for low WMC participants. Firstly, the difference between low WMC and high WMC participants emerged during the third block of trials, precisely during the hunch period as identified in the literature [2,3,4]. Secondly, it appears that a WM load applied during the hunch period of the task impairs subsequent decision making, suggesting that a WM load may prevent the development of hunches [16]. A third clue comes from investigations using the galvanic skin response (GSR), an electrophysiological measure identified as a correlate of hunches [3,4]. Hinson and colleagues observed that a WM load not only leads participants to adopt less advantageous decision patterns on the IGT, but this impairment in IGT decision patterns is also associated with a lack of anticipatory GSR [14,15]. This observation suggests that a limitation in available WM resources may be directly detrimental to the emergence of hunches.Another possible explanation is that the disadvantageous decision pattern of low WMC participants is simply due to a difficulty learning or remembering the outcomes of the various options. Several authors have proposed that participants solve the IGT using memory sampling [16]. In this view, participants would periodically retrieve a few memories of the previous outcomes of each deck, and use these sample memories to guide their next choice. This hypothesis is supported by the observation that certain amnesiac patients fail the IGT, even though there is no reason to suppose that these patients have impaired emotional processing [30]. If memory for previous outcomes is the basis for advantageous decision making in the IGT, then the complexity of the schedule of gains and losses involved in the task may pose a problem to low WMC participants [18]. Indeed, WM is directly related to the ability to selectively retrieve items in long-term memory [31]; low WMC participants may have difficulties sampling their memories of previous outcomes to guide decision making. WM is also related to fluid intelligence [32], which in turn is a predictor of learning rate; if low WMC participants are slower to learn, they may simply need more time to memorize enough outcomes for each option to help their decision making.

There are a few lines of studies that could help disentangle these two hypotheses. A first solution would be to increase the total number of trials in the IGT. If the disadvantageous decision pattern of low WMC participants is caused by a phenomenon of delayed learning, then increasing the number of trials may allow these participants to develop a tendency towards advantageous options, and eventually perform as well as high WMC participants. Future studies may also be interested in combining the individual differences approach with the GSR procedure; this would be an efficient way to check whether WMC is related to the emergence of hunches. If low WMC participants do not develop hunches over the course of the task, then a lack of anticipatory GSR should be observed in these participants [4].

As a conclusion, the individual differences approach seems to be a promising way to investigate the nature of processes involved in the IGT, including working memory. While the IGT has been conceptualized by some authors to be a distinct measure of affective behavioral regulation [33,34] and to capture the affective cognitive processes involved in decision-making [21], our study shows that high-level cognitive functions such as working memory may be strongly involved in complex decision making.
